# Direct evidence of nonstationary collisionless shocks in space plasmas

**DOI:** 10.1126/sciadv.aau9926

**Published:** 2019-02-27

**Authors:** Andrew P. Dimmock, Christopher T. Russell, Roald Z. Sagdeev, Vladimir Krasnoselskikh, Simon N. Walker, Christopher Carr, Iannis Dandouras, C. Philippe Escoubet, Natalia Ganushkina, Michael Gedalin, Yuri V. Khotyaintsev, Homayon Aryan, Tuija I. Pulkkinen, Michael A. Balikhin

**Affiliations:** 1 Swedish Institute of Space Physics, P.O. Box 537, SE-751 21 Uppsala, Sweden.; 2 Department of Earth Planetary and Space Sciences, University of California, Los Angeles, Los Angeles, CA 90095, USA.; 3 Department of Physics, University of Maryland, College Park, MD 20742, USA.; 4 LPC2E, CNRS-University of Orleans, Orleans, France.; 5 Space Sciences Laboratory at University of California, 7 Gauss Way, Berkeley, CA 94720, USA.; 6 Department of Automatic Control and Systems Engineering, University of Sheffield, Sheffield, UK.; 7 Imperial College London, London SW7 2AZ, UK.; 8 IRAP, Université de Toulouse, CNRS, UPS, CNES, Toulouse, France.; 9 European Space Agency/European Space Research and Technology Centre (ESA/ESTEC), Noordwijk, Netherlands.; 10 Finnish Meteorological Institute, Helsinki, Finland.; 11 University of Michigan, Ann Arbor, MI 48109, USA.; 12 Department of Physics, Ben-Gurion University, Beer-Sheva, Israel.; 13 NASA Goddard Space Flight Center, Greenbelt, MD 20771, USA.; 14 Department of Electronics and Nanoengineering, School of Electrical Engineering, Aalto University, Espoo, Finland.

## Abstract

Collisionless shocks are ubiquitous throughout the universe: around stars, supernova remnants, active galactic nuclei, binary systems, comets, and planets. Key information is carried by electromagnetic emissions from particles accelerated by high Mach number collisionless shocks. These shocks are intrinsically nonstationary, and the characteristic physical scales responsible for particle acceleration remain unknown. Quantifying these scales is crucial, as it affects the fundamental process of redistributing upstream plasma kinetic energy into other degrees of freedom—particularly electron thermalization. Direct in situ measurements of nonstationary shock dynamics have not been reported. Thus, the model that best describes this process has remained unknown. Here, we present direct evidence demonstrating that the transition to nonstationarity is associated with electron-scale field structures inside the shock ramp.

## Teaser

Collisionless shocks are ubiquitous throughout the universe, and we present the first direct observation of their nonstationarity.

## INTRODUCTION

As a fluid or plasma flow encounters an obstacle, a shock wave forms if the flow velocity relative to the obstacle exceeds the maximum wave speed in the flow medium. In dense plasmas (e.g., gas binaries), particle collisions are responsible for the transfer of kinetic to heat energy, but in heliospheric space plasmas, the mean free path is too long (~1 astronomical unit) for collisions to play a role. For this reason, it was originally disputed whether shocks in collisionless plasmas could exist at all (*1*). Despite this early debate, collisionless shocks were theoretically predicted (*2*, *3*) and later observed in laboratory (*4*) and space plasmas (*3*, *5*, *6*). We now know that the interplay between electromagnetic field structures and waves plays the role of collisions (anomalous processes, e.g., anomalous resistivity) through thermalization of the plasma, as well as accelerating particles.

The range of spatial scales at which collisionless shocks occur is vast, from millimeters in the laboratory (*7*) to scales of mega parsecs near galaxy clusters (*8*, *9*). Collisionless shocks exist close to many astrophysical objects in the universe and are known to be very efficient in accelerating charged particles. Current thinking suggests that collisionless shocks formed around supernova remnants accelerate ions up to 10^18^ eV (*10*). For these objects, physical information about the system is deduced from observations of emissions from particles that were thermalized or accelerated at collisionless shocks formed near these objects. To date, the only collisionless shocks that can be measured in situ are those in the heliosphere, most notably the terrestrial bow shock, formed when the solar wind plasma encounters Earth’s magnetic field.

The principal characteristic of a collisionless shock is the Mach (*M*_A_) number. With increasing Mach number, collisionless shocks become more efficient accelerators. To maintain a stable shock front, the process of wave steepening must be arrested by some process occurring within the shock front to prevent overturning. In practice, this is achieved by multiple energy transfer mechanisms, which operate over different scale lengths. Notably, these mechanisms manifest as a function of the shock Mach number, leading to the definition of critical Mach numbers, which describes when these mechanisms operate. Most relevant to this study is the whistler critical Mach number (*M*_w_) (*11*), since it is the highest value in which whistler wave precursors can phase stand in the upstream flow. Examples of upstream whistler precursors are abundant in the existing literature (*12*–*14*) and have also shown that nonlinear whistler waves can be the result of plasma instabilities even when the Mach number exceeds *M*_w_. Theory, simulations, and indirect observations suggest that shocks become nonstationary when the Mach number increases above some critical value, leading to cyclic reformation (*15*–*18*). However, since no direct observations have been reported, this process is poorly understood and remains a major unsolved problem of high Mach number shock physics.

The shock surface geometry is also important since it dictates the manner in which plasma is processed by the shock front. It is defined by θ_bn_, the angle between the surface normal and the interplanetary magnetic field direction. A shock is deemed quasi-parallel (quasi-perpendicular) when θ_bn_ is less (greater) than 45°. At Earth, and many other planets, the quasi-perpendicular bow shock magnetic profile typically consists of well-defined foot, ramp, and overshoot regions (*11*). Having said that, the ever-increasing resolution of magnetic field measurements reveals the finer structure of the magnetic ramp (*19*, *20*).

For stationary shocks, the steepening of the magnetic field is balanced by anomalous resistivity, dispersion, or dissipation associated with particle reflection (*11*, *21*). Since the 1980s, it has been theoretically predicted and shown in laboratory plasmas (*15*) that as the Mach number increases, this balance breaks down and the shock front becomes nonstationary and undergoes quasi-periodic reformation (*16*, *17*). Shock front nonstationarity can be characterized as a regime of the shock when nonlinear steepening of the shock front cannot be counterbalanced by other physical processes in a stationary regime, and a quasi-periodic ramp reformation takes place. Before the observations presented in this study, shock front nonstationarity had been observed in numerical simulations (*17*), and only indirect evidence (*22*) had been found in decades of previous in situ observations. To directly confirm a nonstationary shock front requires the ability to resolve the relevant scales governing this process at the time and location where nonstationarity is initiated. Previous investigations have either used other parameters (*22*) or inferred the process from single spacecraft measurements (*23*). Although there are reports (*24*) of dynamic electron-scale structures within the ramp, they may hint toward shock nonstationarity but were not attributed to the process. While these reports are very important, they are all indirect demonstrations of nonstationarity. In this study, we show measurements recorded by two successive spacecraft of the same shock ramp, thus directly observing the magnetic field structures associated with shock nonstationarity.

The gradient catastrophe (GC) model (*16*, *17*) is founded on a dispersive model of weak shocks (*25*) but extended to sufficiently high Mach numbers so that whistler waves are no longer able to phase stand in the upstream flow. Rigorous mathematical analysis of the fundamental set of these model equations, which dictate whistler wave dispersion, and the type of hydromagnetic nonlinearity (advection) concluded that when the shock profile reaches some critical slope/gradient threshold, the shock front becomes unstable and a GC (wave breaking) occurs (*26*). It was proposed that the critical gradients required for the instability to initiate a GC occur when the upstream flow velocity exceeds the velocity of finite amplitude whistler waves (*16*, *17*). The main characteristic of the GC model (*16*, *17*) is the formation of dynamic structures within the shock ramp, which have spatial scales corresponding to the electron dispersion scale, namely, the electron inertial length.

The presence of nonstationary small scales (of the order of the electron inertial scale or below) is a signature of the GC model. Mathematically, a GC is described as the steepening of the profile to an infinite slope with subsequent breaking down of the profile. However, infinite slopes do not exist in nature. The manifestation of the evolution to oversteepening and breaking in the magnetic profile would be the appearance of the smallest possible coherent features, which correspond to the electron-scale limit of the whistler branch. This is the initial stage of the ramp breaking up in two subramps, as implied by the GC model.

Nonstationarity can lead to shock reformation; however, it should be noted that although a reformed shock must have been nonstationary, a nonstationary shock does not have to lead to reformation. Therefore, the mechanism in which nonstationarity is initiated in the GC model is by nature a local phenomenon. The global restructuring of the shock may occur later and not in concert with the GC. Thus, the global-scale effects on the shock structure may not be obvious until later when or if reformation takes place.

To experimentally confirm the GC model, the observations must have the capability to resolve the smallest spatial scales intrinsic to these models. The difficulty of this task owes to the fact that the spatial scales of magnetic structures in the GC (*16*, *17*) model are close to the range of typical electron scales, which require the simultaneous measurement by two or more spacecraft, separated by small distances (a few kilometers in the case of the terrestrial bow shock). Until recently, these measurements were not possible. To address this problem, the European Space Agency Cluster mission commenced a campaign to achieve shock observations with extremely small inter-spacecraft separation distances. We report a clear agreement between our observations and the GC model and, therefore, provide direct measured evidence of shock front nonstationarity at the theoretically predicted scales. This has provided a unique opportunity to study the magnetic and electric field structures, which are fundamental to the dynamics of shock front nonstationarity. We present the results from these unique observations in this letter.

## RESULTS

The Cluster mission consists of four identical spacecraft launched in July and August 2000. To resolve spatial and temporal ambiguities over multiple scales, the separation between the probes has varied between tens of kilometers to more than 100,000 km. Designed to resolve mesoscale structures, the majority of observations are not suitable for observing shock nonstationarity since the spacecraft separation did not attain the required electron scales. However, at the last stage of the mission, the special close separation campaign positioned two probes within a few kilometers of each other (see fig. S2), which at the terrestrial bow shock is typically on the same order as the electron inertial length. This inter-spacecraft separation means that it is sufficient to resolve electron-scale structures, which are associated with the initiation of shock front nonstationarity according to the GC model (*16*, *17*).

**Fig. 1:**
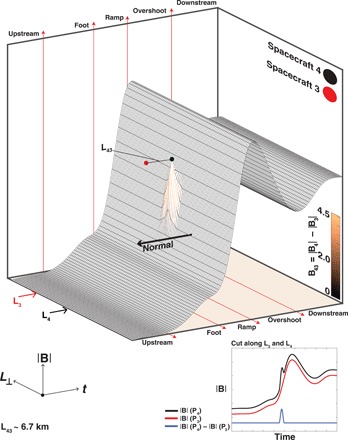
Bow shock ramp substructure. An illustration of the magnetic profile (|**B**|) of the quasi-perpendicular terrestrial bow shock as a function of time and tangential (*L*_⊥_) distance along the shock surface. The profile shows the following regions: upstream/solar wind, shock foot, ramp, overshoot, and the downstream/magnetosheath. The time series panel corresponds to cuts of artificial data in the plane perpendicular to the shock normal along L_3_ (red) and L_4_ (black). The blue trace represents the difference between the two cuts. It is this difference that is used to shade the shock surface to highlight the substructure location. It is clear that depending on the localized position of the spacecraft (in this case on electron scales), one may observe the structure and another will not. If the separation is too large, both may miss the structure or it will be impossible to correlate simultaneous measurements. In our case, the probe locations were at ideal locations and well suited to resolving the theoretically predicted scales.

Figure 1 represents in the form of an illustration the shock front surface and an electron-scale structure embedded into the ramp. Since the shock motion is along the normal, the probes traverse the shock (from solar wind to magnetosheath) along the two directions indicated by L_3_ and L_4_. The cuts of artificial magnetic field profiles (offset for visibility) along these directions can be found in the lower panel and show one spacecraft measuring the structure and another not. The blue trace shows the difference between the measurements to highlight the presence of the ramp structure.

**Fig. 2:**
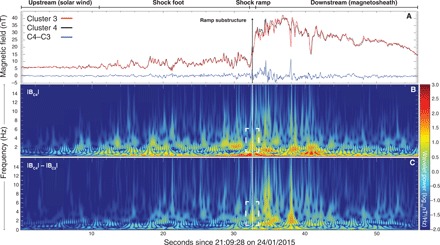
Bow shock wavelet spectrogram. Plotted in (**A**) are the time series of the magnetic field modulus measured by the Clusters 3 (red) and 4 (black) FluxGate Magnetometer instruments during the bow shock traversal. The blue trace shows the difference (4–3) between the measurements. (**B**) Wavelet spectrogram of the C4 bow shock crossing. (**C**) Wavelet spectrogram but computed with the difference data. The vertical lines in each panel indicate the location of the electron-scale substructure, which is the focus of this paper. There is a clear enhancement of wavelet power at the times corresponding to this structure (white dashed boxes). Since the difference data plotted in (C) have the shock ramp removed, then it is the direct evidence of an electron-scale ramp substructure within the ramp. For more evidence that this is associated with a nonstationary shock front, we provide a more detailed view of the shock crossing and the three-dimensional evolution of the magnetic field components during the ramp substructure in Fig. 3.

Figure 2 presents magnetic field measurements (|**B**|) from 24 January 2015 when Cluster 3 (C3) and Cluster 4 (C4) crossed the terrestrial bow shock. For additional details and the criteria of the event selection process, see the Materials and Methods section. In Fig. 2A, the red, black, and blue traces correspond to the magnetic field time series of C3, C4, and their difference (B_43_ = |**B**_4_| − |**B**_3_|), respectively. Both spacecraft measured the shock ramp 32 s into the interval almost simultaneously due to the 7-km proximity of the probes. Noteworthy in these observations are the small dissimilarities within the ramp, which are indicative of electron-scale differences between the spacecraft observations during the shock ramp. Figure 2 (B and C) illustrates the spectral features of the C4 magnetic field data and the difference B_43_, respectively. In Fig. 2C, during the shock ramp (vertical line), there are enhancements in the wavelet spectrum that was computed using B_43_ (boxed region). If two spacecraft with exactly the same instrumentation are at the same point, then B_43_ should be zero and the dynamical spectrum calculated will be absent of features above the noise level. The feature located at frequencies between 2 and 4 Hz is therefore indicative of the presence of phenomena within the shock ramp whose spatial scale can be derived based on the inter-spacecraft separation. This demonstrates that during the shock ramp, an electron-scale substructure is present in accordance with the 7-km inter-spacecraft separation. It is also noteworthy that other differences between C3 and C4 are observed downstream. However, after analyzing these structures, they do not appear to be of a similar nature to the one measured during the shock ramp, and therefore, the focus of this study remains strictly on the measurements during the shock ramp region.

**Fig. 3:**
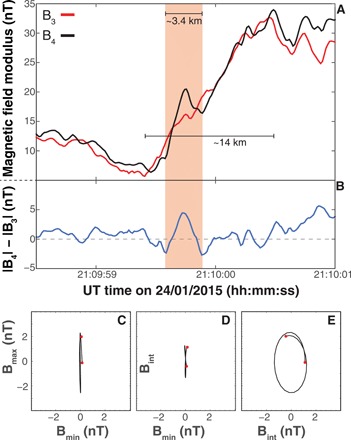
Evidence of electron-scale subshock structure. (**A**) The magnetic field modulus of the bow shock crossing by C3 (blue) and C4 (red). The difference (|**B**_4_| − |**B**_3_|) is plotted in (**B**). The red shaded region marks a large difference in the shock ramp between C3 and C4. The structure in (B) is evidence that the shock ramp substructure varies over electron scales, because features in this dataset are dictated by the inter-spacecraft separation of 7 km. We have marked the spatial length scales of the ramp and the substructure (see the Materials and Methods section for how these lengths were derived). (**C** to **E**) Hodograms derived from the application of minimum variance analysis to the magnetic field differences in the shock frame. The variations appear elliptically polarized and propagate almost parallel to the shock normal, consistent with whistler waves on the predicted scales of the GC model of shock front reformation (*17*) (see the Materials and Methods section for technical details of the hodograms). UT, Universal Time.

An expanded time scale of |**B**| measured by C3 (red) and C4 (black) is plotted in Fig. 3A. Here, the ramp substructure is visible, especially from the difference (B_43_ = |**B**_4_| − |**B**_3_|) plotted in Fig. 3B below. These data are equivalent to the sketched profile shown in Fig. 1. While similar nonmonotonic features and electric field spikes within the ramp have previously been reported (*27*) in Cluster data, the spacecraft separation was larger, and the electron-scale structures appeared uncorrelated. As a result, neither the spatial scales of these features nor their correspondence to any particular shock front nonstationarity model could be determined. These unique observations with short electron-scale separation of the spacecraft prove that the observed features are substructures of the ramp and exhibit spatial scales of the order of the electron inertial length *c*/ω_ρe_. Furthermore, the angle (θ_kn_) measured between the minimum variance direction of B_43_ ($\hat{k}$) and the shock normal $\hat{n}$ is less than 6°, consistent with the GC model. The hodograms in Fig. 3 (C to E) demonstrate that the evolution of B_43_ is similar to an elliptically polarized wave, indicating that the field rotates around the direction almost parallel to the shock normal. We refer the reader to the Materials and Methods section for technical details on the calculation of the shock normal, shock parameters, and the hodograms. The occurrence of these oscillations within the ramp points toward the onset of a GC (*16*, *17*). It is this oscillatory feature measured exactly within the ramp that makes these observations consistent with the GC shock front reformation model (*16*, *17*), in which the transition to nonstationarity is initiated within the ramp.

**Fig. 4:**
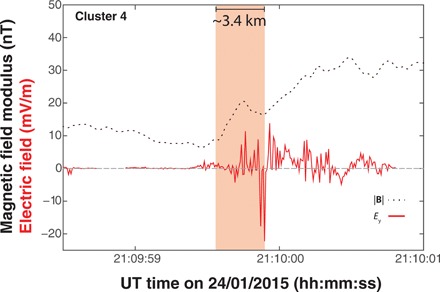
C4 Bow shock electric field on 24 January 2015. Indicated by the red trace is the electric field (*E*_y_) measured by the C4 EFW instrument in the spacecraft frame. The start of the large oscillations marks the start of the shock transition region. The dotted black trace is the modulus of the magnetic field for reference. The large amplitude *E*_y_ variations occur in concert with the magnetic structure on the shock ramp indicated by the shaded red region. Note that the electric field structures take place on electron scales, which are theoretically predicted by the model of Krasnoselskikh *et al*. (*17*).

The red trace plotted in Fig. 4 shows the spacecraft frame electric field (*E*_y_) measured by the C4 EFW (Electric Field and Wave) instrument during the shock traversal. Unfortunately, electric field measurements from C3 were unavailable for comparison during this time. For reference, |**B**| has also been included (dotted black trace). The shock crossing is noticeable in *E*_y_ by the sudden increase in amplitude of fluctuations (>25 mV/m) within the ramp and then a continuation of subsequent variations downstream. Note the presence of electron-scale fluctuations in *E*_y_ in the vicinity to the occurrence of the ramp substructure. The size of this structure is approximately 3.4 km compared with the ramp length of approximately 14 km (*c*/ω_ρe_ ~1.6 km). The calculation of these scales is provided in the Materials and Methods section.

## DISCUSSION

In low Mach number quasi-perpendicular dispersive shocks, the steepening of the shock front will cease when the shock front length approaches the whistler dispersion scale (*11*), but only if the shock front Mach number remains lower than the whistler critical Mach number. In that case, a phase standing precursor will form to provide the dissipation. However, when *M*_A_ > *M*_w_, steepening continues, and nonlinear whistler waves develop within the ramp. This leads to the development of small-scale features within the ramp and nonstationarity. Since the maximum phase velocity of whistler waves corresponds to scales *c*/ω_ρe_, then the ratio of the electric to magnetic field of the wave will increase as one approaches these scales. These features within the ramp can be observed in both magnetic and electric field measurements and have characteristic spatial scales of *c*/ω_ρe_. Although not directly addressed in this work, these structures may lead to nonadiabatic electron thermalization (*28*). When *M*_A_ > *M*_w_, nonstationarity can be initiated; however, if the Mach number exceeds the nonlinear whistler critical Mach number $\left(M_{\text{nW}}=\sqrt{2M_{W}}\right)$ (*17*), then the development of a GC occurs, nonstationarity is initiated, and ramp reformation is the only possible outcome. For the shock studied here, the parameters (*M*_A_ = 9.4, *M*_w_ = 8.7 and *M*_nW_ = 12.3) are ideal for the development of nonlinear whistler waves within the shock ramp, indicating the transition to a nonstationary regime.

One more possible candidate to explain these observed shock front substructures is shock front rippling. However, the observed features cannot represent shock front rippling since the spatial scales of “ripples” should be much larger on the order of typical ion plasma lengths (*20*, *29*).

Nonstationarity has also been observed in particle in cell (PIC) (ions and electrons are particles) and hybrid (ions are treated as particles, while electrons as a fluid) numerical simulations (*30*, *31*). It is interesting to note that in our case, the observations agree with the GC model since nonstationarity is initiated inside the ramp and not upstream of the shock ramp. A possible explanation for this could be the different parameter regimes (e.g., the ratio of the plasma frequency to the electron cyclotron frequency) of our case study and the PIC and hybrid model runs (*32*), which make a direct comparison difficult. However, this does not confirm or deny the validity of the underlying physical mechanisms in other models leading to nonstationarity, although both mechanisms are mutually exclusive in the context of a single shock crossing. It is logical to conclude that in nature, the physical mechanism controlling the nonstationarity of a collisionless shock front may vary depending on the plasma regime in which it occurs.

Observations have shown that higher frequency waves can have accompanying large (100s of mV/m) amplitude electric fields (*19*). However, these higher-frequency waves do not have the same efficiency for ion reflection and are therefore not applicable as a mechanism to explain the initiation of nonstationarity at the terrestrial bow shock. It should also be noted that features within the ramp have been previously reported (*33*) in simulations. In these cases, their spatial scales were on the order of ion scales.

The shock presented here is in the exact regime where waves formed within the ramp and exhibits (Fig. 4) electron-scale field structures in full agreement with the process theoretically predicted by the GC model (*16*, *17*); we observe a clear electron-scale whistler wave inside the ramp with accompanying electric field spikes when the shock is in the correct parameter regime. During the initiation of nonstationarity, a GC occurs when whistlers can no longer stand, suggesting the dynamic development of a whistler wave that will further escape downstream. We directly observed the generation of such a whistler inside the ramp. Since the developing structure is on the order of electron scales, it is a clear signature of the GC model and nonstationarity. Further support of this is provided by the fact that the minimum variance direction of the observed substructure is almost aligned with the shock normal direction, as predicted by the theoretical GC model (*16*, *17*). It is noteworthy that other electron-scale structures can arise from electron dynamics. However, because of the clear agreement with the GC model, this was the most plausible outcome. Nevertheless, it will be important to analyze additional shock crossing with similar and different features to construct a more complete understanding on their role in regulating the global shock structure.

We present direct measured evidence of a case where the initiation of a nonstationarity collisionless shock front occurs within the ramp and on electron scales theoretically predicted by the GC model (*16*, *17*). These findings not only provide a key missing link for collisionless shock research but also contribute to understanding physics in other plasma environments where particle heating takes place on kinetic scales, and to the fundamental physics of systems that experience energy transport across multiple scales. Examples of relevant plasma environments include the Sun (e.g., coronal heating), planetary environments and the solar wind (e.g., magnetic reconnection and plasma turbulence), and astrophysical plasmas (e.g., galactic jets).

## MATERIALS AND METHODS

### Event selection

A detailed survey was performed of the shock crossings, which took place during the Cluster close separation campaign between 28 December 2014 and 5 February 2015. For a suitable candidate, at a minimum, the following criteria had to be met: (i) The shock crossing had to be in the correct parameter regime to exhibit nonstationary dynamics; (ii) the shock geometry had to be quasi-perpendicular (θ_bn_ > 45^o^); (iii) the spacecraft position on the shock layer had to be suitable to measure nonstationary structures; and (iv) the ability to determine an accurate shock normal.

### Calculation of bow shock parameters

The bow shock normal was determined using the model of Peredo *et al*. (*34*) and yielded a direction of $\hat{n}=[0.98,0.21,0.05]$ in the Geocentric Solar Ecliptic frame. This direction was validated by projecting the magnetic field along the shock normal $\left(B_{n}=B∙\hat{n}\right)$ and ensuring that no significant variations were present across the shock ramp; this is demonstrated in fig. S1. The resolution of the magnetic field data used in this study is 66.6 Hz. To calculate the shock geometry θ_bn_, a 30-s duration of the upstream magnetic field was used, **B_0_** = [3.41, −4.63, 0.96] nT, which produced a geometry of θ_bn_ = 66°. In addition, the difference between **B_0_** computed from C3 and C4 was less than 0.5°. The Alfvén Mach number *M*_A_ of the shock was calculated from the ratio between the upstream normal flow speed projected along the shock normal direction $\left(V_{n}=V_{\text{SW}}∙\hat{n}=315\text{km}/s\right)$ and the local Alfvén velocity ($V_{A}=\sqrt{B_{0}^{2}/\mu_{0}\rho }=33.4$ km/s), which gave *M*_A_ = 9.4, where μ_0_ is the permeability of free space and ρ is the mass density (*m*_i_*n*_i_). The ion density was calculated using the electron plasma frequency measured by the WHISPER instrument. For this calculation, we took the maximum of the plasma frequency cutoff from the WHISPER spectrogram upstream of the shock foot. Since this varied from 33 to 35 kHz, we adopt the intermediate value of 34 kHz. A value of *f*_ρe_ = 34 kHz yields (for a quasi-neutral proton plasma in which *n*_e_ ~ *n*_i_), a proton density of 14.3 cm^−3^. The upstream flow velocity was determined directly from the CIS (Cluster Ion Spectrometry) particle distributions, which gave **V**_sw_ = [−325, 0, 50] km/s. This velocity was carefully calculated by considering only particles arriving in the solar wind direction, ±45° in azimuth, thus eliminating any contributions from particles moving antiparallel to *V*_x_ and also “ghost” signals due to crosstalk from particles arriving from the opposite direction (to *V*_x_), which reduces the absolute value of *V*_x_. The linear critical whistler Mach number was calculated from $M_{w}=|\text{cos}\theta_{\text{bn}}|/2\sqrt{\mu }$, where μ is the electron-to-proton mass ratio. The nonlinear critical Mach number (when a GC occurs) was calculated from $M_{\text{nW}}=\sqrt{2M_{w}}$.

### Shock velocity determination

To separate spatial and temporal ambiguities, it is essential that the velocity of the shock front is determined. The shock velocity was calculated from $V_{\text{sh}}=R_{\text{sep}}∙\hat{n}/t_{\text{sep}}$, where the subscript sep refers to the separation (distance and time) of two spacecraft. However, for this particular shock crossing, this was a nontrivial task. Errors can be large when two spacecraft are closely separated; therefore, the pairing of C3 and C4 could not be used. The pairings of C_41_ and C_42_ were also suboptimal and prone to error as their separation vectors are almost perpendicular to $\hat{n}$, giving θ_41n_ = 87.4° and θ_42n_ = 89.5°, respectively. Therefore, we used C_12_, in which R_12_ = 3688 km and θ_R12n_ = 78.7°, which provided *V*_sh12_ = 9.2 km/s. For comparison, the shock velocity determined using C_14_ was *V*_sh14_ = 9.5 km/s and is very close to the estimate from the C_12_ pair. To be thorough, we also used a separate method, which was based on the width of the foot region (*35*) and was calculated from *L*_foot_ ≈ 0.68 sinθ_bn_(*V*_SW_/Ω_ci_). This method results in a value of *V*_sh_ ≈ 14 km/s, which agrees with the previous estimates. Because of the unfavorable spacecraft geometry, the shock velocity determined from the foot was used in the present manuscript. However, using the C_12_ and C_14_ speeds would not affect the outcome from this study since the size of the observed structures would remain on the order of electron scales.

### Spatial scale of the substructure

The duration of ramp substructure according to B_43_ is *t*_sub_ = 0.24 s. On the basis of *V*_sh_ = 14 km/s, this corresponds to a spatial scale of *R*_sub_ = *V*_sh_*t*_sub_ = 3.4 km, assuming a negligible spacecraft velocity. The upper limit on the spatial scale (in the plane perpendicular to $\hat{n}$) of the substructure can be estimated using the component of the C_34_ separation vector that is perpendicular to the normal because the substructure is prominent in C_4_ data but not C_3_. This suggests *E*_sub⊥_ ≤ 4.8 km. The estimates correspond to approximately 2.1 and 3 electron inertial lengths *c*/ω_ρe_ = 1.6 km. For comparison, the ion inertial length is *c*/ω_ρi_ = 68.7 km, which is an order of magnitude above the spatial scale of the structures we measured.

### Hodograms

Here, we describe the method for obtaining the hodograms plotted in Fig. 3 (C to E). First, we perform minimum variance analysis on the substructure consisting of 30 data points. The minimum variance direction is determined from the eigenvectors of the covariance matrix calculated from the magnetic field data of the substructure. The ratios of the eigenvalues for each eigenvector can be used as a proxy for how well determined the minimum variance directions are. Each eigenvector corresponds to the direction of minimum, intermediate, and maximum magnetic field variance, and thus by plotting each combination of these [*B*·*E*_(min,int,max)_] against each other, one can obtain a hodogram as shown in Fig. 3. The hodogram represents the evolution of the magnetic field in each of these planes. Considering each panel, the polarization can be inferred, and in this case, the structure appears to be elliptically polarized. Before implementing the minimum variance analysis, we applied a band-pass filter to remove any other contaminations, as shown previously to improve the accuracy of this analysis (*36*, *37*). In this case, we removed frequencies below 2 Hz and above 4 Hz. This produced eigenvalues of λ_min,int,max_ = [0.0029, 0.7760, 2.7813]. Note that, although this improves the result, reducing or expanding these cutoff frequencies does not alter the conclusions, and thus, the result here is quite robust.

## Supplementary Material

http://advances.sciencemag.org/cgi/content/full/5/2/eaau9926/DC1

## SUPPLEMENTARY MATERIALS

Supplementary material for this article is available at http://advances.sciencemag.org/cgi/content/full/5/2/eaau9926/DC1 Fig. S1. C3 bow shock normal. Fig. S2. C1 to C4 bow shock crossings. Fig. S3. Cluster spacecraft constellation.
