# Supplementary material for: Direct evidence of nonstationary collisionless shocks in space plasmas
Source: Sci Adv. 2019 Feb 27;5(2):eaau9926. doi: 10.1126/sciadv.aau9926 (PMC6392793; doi:10.1126/sciadv.aau9926)
Supplement: http://advances.sciencemag.org/cgi/content/full/5/2/eaau9926/DC1 [file supp_5_2_eaau9926__index.html]

Science Advances | Science Advances

## Supplementary Materials

**This PDF file includes:**

- Fig. S1. C3 bow shock normal.
- Fig. S2. C1 to C4 bow shock crossings.
- Fig. S3. Cluster spacecraft constellation.

Download PDF

**Files in this Data Supplement:**

- Adobe PDF - aau9926\_SM.pdf
