# Supplementary material for: Direct evidence of nonstationary collisionless shocks in space plasmas
Source: Sci Adv. 2019 Feb 27;5(2):eaau9926. doi: 10.1126/sciadv.aau9926 (PMC6392793; doi:10.1126/sciadv.aau9926)
Supplement: http://advances.sciencemag.org/cgi/content/full/5/2/eaau9926/DC1 [file aau9926_SM.pdf]

Science Advances
AAAS

advances.sciencemag.org/cgi/content/full/5/2/eaau9926/DC1

Supplementary Materials for

Direct evidence of nonstationary collisionless shocks in space plasmas

Andrew P. Dimmock*, Christopher T. Russell, Roald Z. Sagdeev, Vladimir Krasnoselskikh, Simon N. Walker, Christopher Carr, Iannis Dandouras, C. Philippe Escoubet, Natalia Ganushkina, Michael Gedalin, Yuri V. Khotyaintsev, Homayon Aryan, Tuija I. Pulkkinen, Michael A. Balikhin

*Corresponding author. Email: andrew.dimmock@irfu.se

Published 27 February 2019, *Sci. Adv.* **5**, eaau9926 (2019)
DOI: 10.1126/sciadv.aau9926

This PDF file includes:

Fig. S1. C3 bow shock normal.
Fig. S2. C1 to C4 bow shock crossings.
Fig. S3. Cluster spacecraft constellation.

Supplementary Materials

Bow shock normal

Figure S1 shows the magnetic field measured by Cluster 3 (black trace) during the shock transition. The red vertical lines mark the interval corresponding to the ramp region. The blue trace corresponds to the normal component of the magnetic field (B_n), which is determined by projecting the magnetic field along the shock normal direction. For an accurate shock normal direction, little to no variation (or offset) of B_n should take place during the shock ramp. It is clear from the blue trace that between the vertical red lines, B_n is almost unchanged across the ramp indicating an accurate shock normal direction.

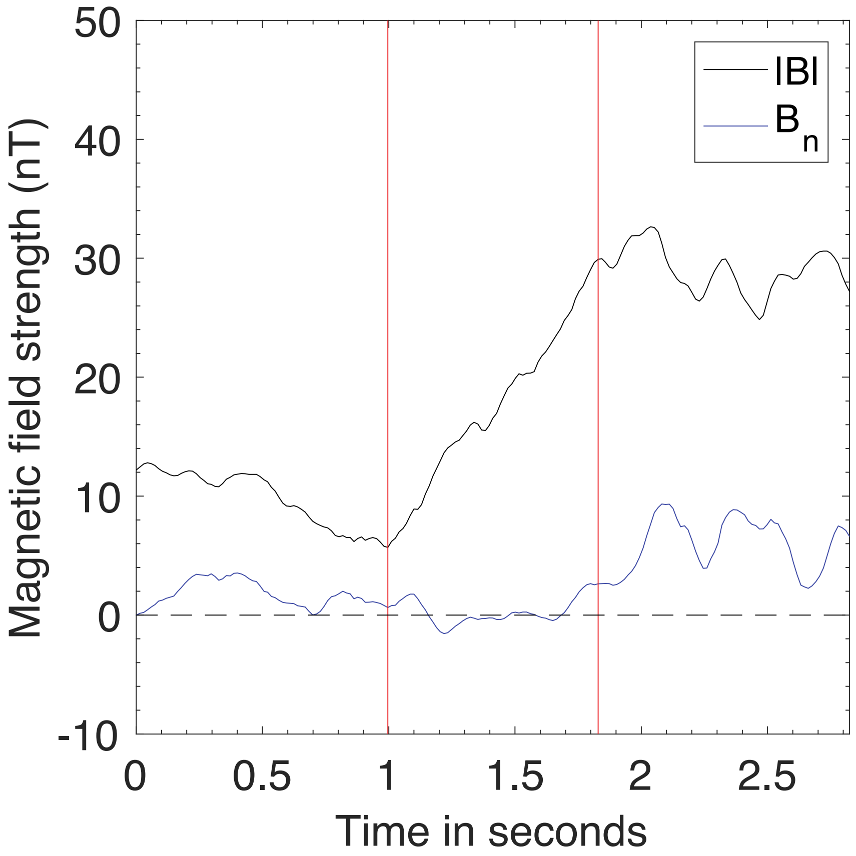

Fig. S1. C3 bow shock normal. The magnetic modulus of Cluster 3 (black trace), and the shock normal component (blue trace). The vertical red lines show the shock ramp. It is clear from the normal component that there are no significant variations during the shock ramp, confirming an accurate shock normal direction.

Shock crossings on 24 January 2015

Figure S2 presents the magnetic modulus measured by each of the four Cluster probes on 24/01/2015. The delay of the crossing times in panels (a & b) are due to the 3684 km separation between Cluster 1 and Cluster 2. Because of the Cluster close separation campaign, the almost simultaneous crossings made by Cluster 3 and Cluster 4 are achieved by only a 6.7 km separation. The close proximity allows small scale structures (local electron inertial lengths) to be resolved within the shock layer. This shock crossing is also in the correct parameter regime to study shock nonstationarity and as a result, we were able to identify and resolve an electron scale sub-structure embedded in the ramp. The physical implications of this are described in the main article.

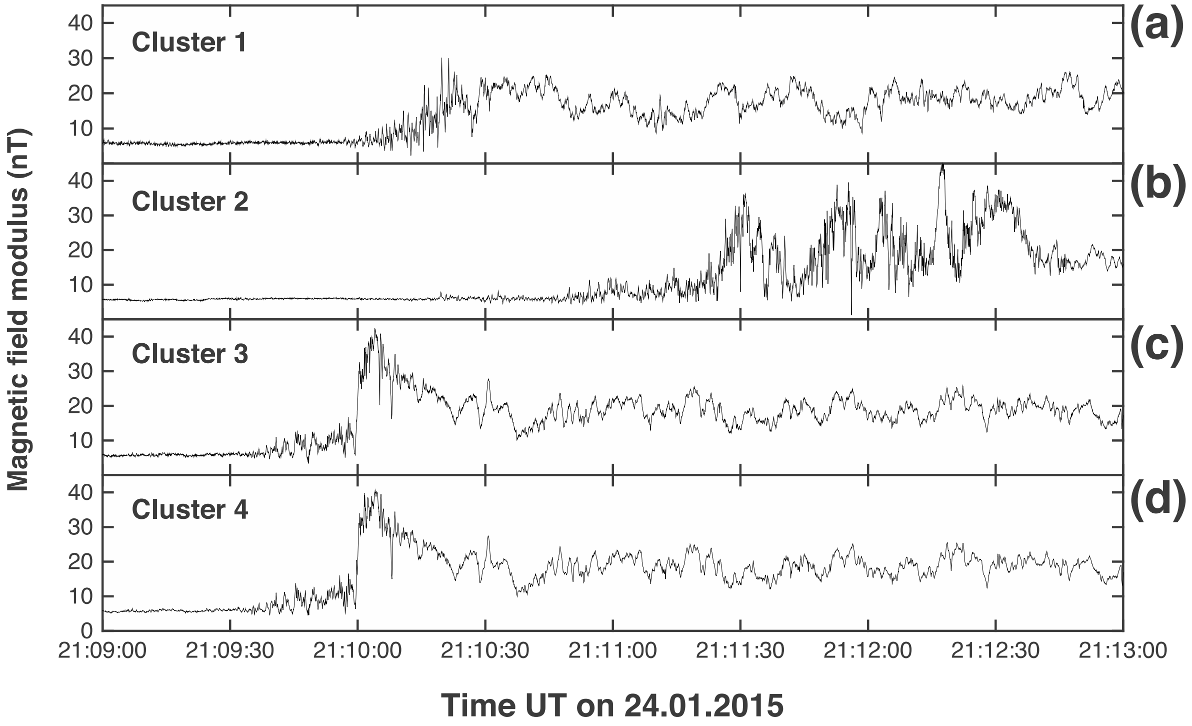

Fig. S2. C1 to C4 bow shock crossings. This figure shows the magnetic field (|**B**|) profile of each bow shock crossing made by Cluster 1-4 (**A** to **D**). The crossings by 1-2 are separated by approximately 60 seconds due to the larger separation of these probes. The 6.7 km separation of Cluster 3 and 4 results in almost simultaneous crossings of the bow shock. The differences between these measurements are the focus of the present study.

Cluster spacecraft constellation on 24 January 2015

Figure S3 shows the configuration of the Cluster spacecraft during the shock crossing shown in figs. S1 and S2. The Cluster close separation campaign placed Cluster 3 and Cluster 4 only 6.7 km apart, significantly closer than the remaining spacecraft pairings. The magnitude of each inter-spacecraft separation vector can be found in the bottom right. This unique configuration has allowed for the first time, the direct observation of a collisionless shock as it transitions to a nonstationary state. This small separation is crucial as it is needed to resolve the required electron scales structures which are associated with this process - which was achieved in this case study.

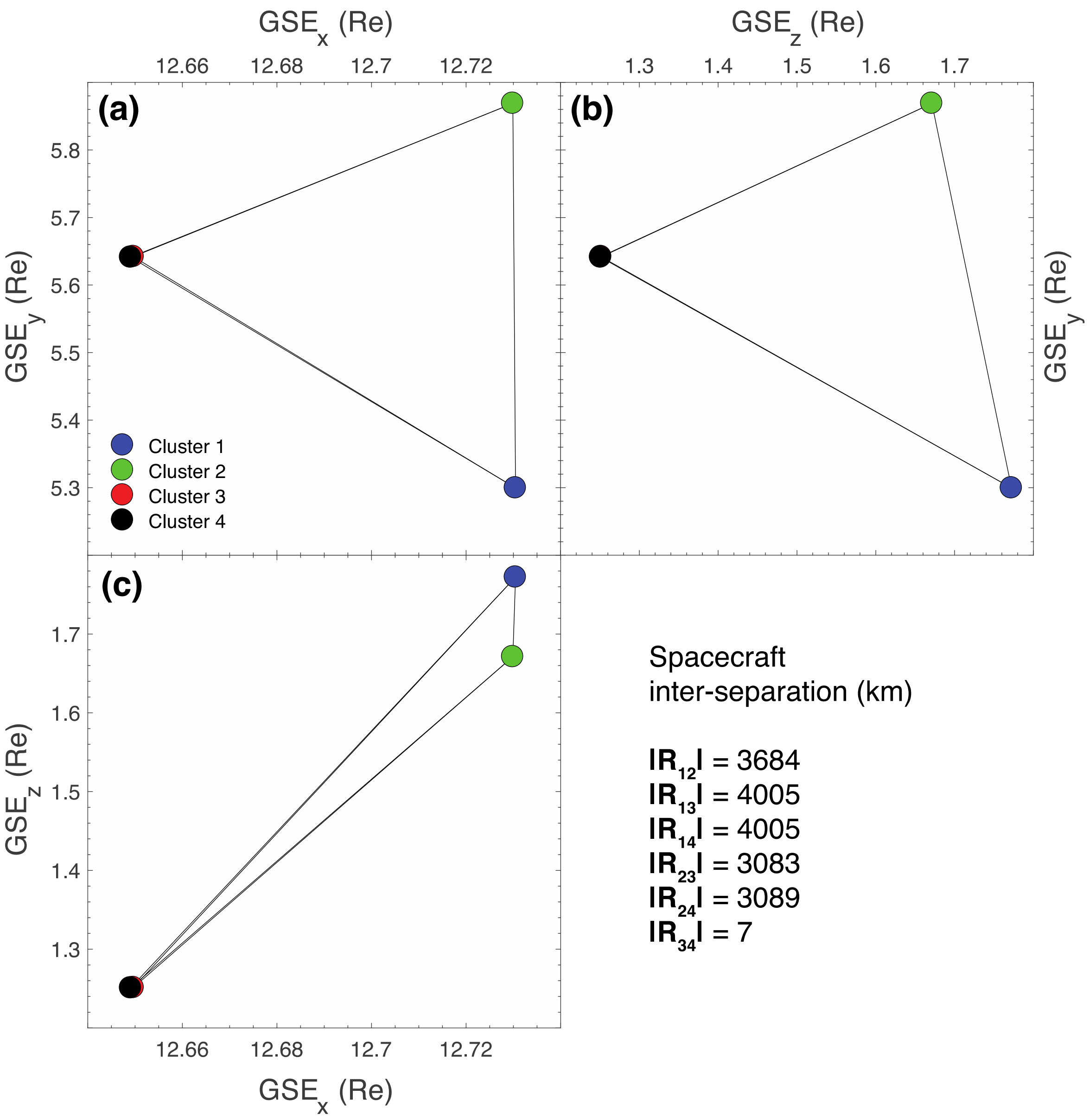

Fig. S3. Cluster spacecraft constellation. Panels (**A** to **C**) show the configuration of the Cluster spacecraft in the XY, XZ and ZY planes. The scale of each axis is Earth Radii which is 6371 km. The magnitude of the separation vectors is listed in the bottom right.
